# Must Reorganize

**Published:** 1916-02

**Authors:** 


### Must Reorganize.

The secular press brings the information that the Supreme Court of Illinois has ordered a writ of mandamus directing the State's attorney of Cook county, Illinois, to bring quo warranto proceedings against the American Medical Association for the removal of the present officers. It is not known whether the quo warranto proceedings will be contested or not, but inasmuch as the decision comes from the Supreme Court of the State it is probable that reorganization will be effected without further contest.

The suit resulting in this decision has been pending five years, and was instituted by Dr. Frank Lydston, of Chicago, who claimed that the officers were illegally elected, and who sought to prevent Dr. George H. Simmons from holding three offices in the Association.

The future course of the court proceedings, should there be any, and the reorganization will be watched with keenest interest by the physicians of the entire country.

